# 201 Assessing medical students satisfaction with a physical activity and health course: results from the VANGUARD European Union Erasmus+ Project implementation in Portugal

**DOI:** 10.1093/eurpub/ckae114.006

**Published:** 2024-09-26

**Authors:** Ana Barbosa, Henrique Barros, Romeu Mendes

**Affiliations:** EPIUnit - Instituto de Saúde Pública, Universidade do Porto, Portugal; Laboratório para a Investigação Integrativa e Translacional em Saúde Populacional (ITR), Porto; EPIUnit - Instituto de Saúde Pública, Universidade do Porto, Portugal; Laboratório para a Investigação Integrativa e Translacional em Saúde Populacional (ITR), Porto; EPIUnit - Instituto de Saúde Pública, Universidade do Porto, Portugal; Laboratório para a Investigação Integrativa e Translacional em Saúde Populacional (ITR), Porto; Unidade Local de Saúde de Trás-os-Montes e Alto Douro, Vila Real

## Abstract

**Purpose:**

The inclusion of physical activity into the undergraduate curricula of healthcare professionals is recommended by the World Health Organization and the European Commission as a key policy to promote health-enhancing physical activity (HEPA).

The VANGUARD project implementation in Portugal, led by the Institute of Public Health of the University of Porto (ISPUP), was supported by the European Union Erasmus+ Programme and had the goal of including physical activity in the academic curricula of healthcare professionals across European nations.

The purpose of this work was to assess the satisfaction of medical students regarding a Physical Activity and Health course implemented in a Portuguese Medical School.

**Project description:**

Students enrolled in the second year (academic year 2022/2023) of the Integrated Master’s Degree in Medicine of the School of Medicine and Biomedical Sciences of the University of Porto (ICBAS), were invited to participate in a Physical Activity and Health course using a self-paced learning method. The course was designed by the ISPUP with the support of the Board of Directors of the Integrated Master in Medicine of ICBAS. It was available for six weeks on an online training platform. The course comprised a total of 26 thematic sessions, continuous evaluation and a final case study. After the course completion, students rated their satisfaction with several features of the course on a 5-point Likert scale (1: very dissatisfied; 5: very satisfied).

Out of the 185 students registered, 46 expressed the desire to participate, 34 joined the course, and 27 successfully completed it. More than half of students were satisfied or very satisfied, respectively, with: online training platform (55.6% and 33.3%); self-paced learning method (33.3%, 51.9%); sessions’ content quality (51.9%, 37.0%); continuous evaluation (48.1%, 29.6%); and the final case study (55.6%, 18.5%). Globally, most students were very satisfied with the course (51.9%).

**Conclusions:**

Medical students revealed an overall good satisfaction with the Physical Activity and Health course, which supports the inclusion of this course in the Integrated Master’s Degree in Medicine. These results may have positive implications for future medical doctors in terms of promoting HEPA among patients and communities.

